# Conditioned medium from stem cells derived from human exfoliated deciduous teeth ameliorates NASH via the Gut-Liver axis

**DOI:** 10.1038/s41598-021-98254-8

**Published:** 2021-09-21

**Authors:** Hisanori Muto, Takanori Ito, Taku Tanaka, Shinya Yokoyama, Kenta Yamamoto, Norihiro Imai, Yoji Ishizu, Keiko Maeda, Takashi Honda, Tetsuya Ishikawa, Asuka Kato, Taichi Ohshiro, Fumiya Kano, Akihito Yamamoto, Kiyoshi Sakai, Hideharu Hibi, Masatoshi Ishigami, Mitsuhiro Fujishiro

**Affiliations:** 1 Department of Gastroenterology and Hepatology, Nagoya University Graduate School of Medicine, 65 Tsuruma-cho, Showa-ku, Nagoya, Aichi 466-8550 Japan; 2 ITOCHU Collaborative Research-Molecular Targeted Cancer Treatment for Next Generation, Nagoya University Graduate School of Medicine, Nagoya, Japan; 3 Department of Tissue Regeneration Institute of Biomedical Sciences, Tokushima University Graduate School, Tokushima, Japan; 4 Department of Oral and Maxillofacial Surgery, Nagoya University Graduate School of Medicine, Nagoya, Japan

**Keywords:** Stem cells, Gastroenterology, Medical research

## Abstract

Non-alcoholic steatohepatitis (NASH) occurrence has been increasing and is becoming a major cause of liver cirrhosis and liver cancer. However, effective treatments for NASH are still lacking. We examined the benefits of serum-free conditioned medium from stem cells derived from human exfoliated deciduous teeth (SHED-CM) on a murine non-alcoholic steatohepatitis (NASH) model induced by a combination of Western diet (WD) and repeated administration of low doses of carbon tetrachloride intraperitoneally, focusing on the gut-liver axis. We showed that repeated intravenous administration of SHED-CM significantly ameliorated histological liver fibrosis and inflammation in a murine NASH model. SHED-CM inhibited parenchymal cell apoptosis and reduced the activation of inflammatory macrophages. Gene expression of pro-inflammatory and pro-fibrotic mediators (such as *Tnf-α, Tgf-β,* and *Ccl-2*) in the liver was reduced in mice treated with SHED-CM. Furthermore, SHED-CM protected intestinal tight junctions and maintained intestinal barrier function, while suppressing gene expression of the receptor for endotoxin, *Toll-like receptor 4*, in the liver. SHED-CM promoted the recovery of Caco-2 monolayer dysfunction induced by IFN-γ and TNF-α in vitro. Our findings suggest that SHED-CM may inhibit NASH fibrosis via the gut-liver axis, in addition to its protective effect on hepatocytes and the induction of macrophages with unique anti-inflammatory phenotypes.

## Introduction

It has been reported that approximately 25% of adult patients have a non-alcoholic fatty liver disease (NAFLD)^1^. Among NAFLD patients, 12–40% develop non-alcoholic steatohepatitis (NASH), which could progress to cirrhosis and hepatocellular carcinoma^2^. The proportion of non-viral (hepatitis B and C virus) hepatitis has been increasing in recent years among the background liver diseases of hepatocellular carcinoma, and is expected to continue to increase in the future^3^. However, there are no established and effective drug therapies for NASH, and the development of new treatments is emerging.

Stem cell therapy is a promising approach for the treatment of NASH. Efficacy has been reported in animal models of steatohepatitis by treatment with bone marrow and adipose-derived stem cells^4,5^. Mesenchymal stem cells (MSCs) with multipotent differentiation potential and low antigenicity self-renew and differentiate into adipocytes, cartilage, and bone, and secrete a wide range of nutritional and immunomodulatory factors. These soluble factors can be collected as serum-free conditioned medium. Recent studies have shown that factors derived from various types of stem cells may potentially treat many incurable diseases^6^. Stem cells from human exfoliated deciduous teeth (SHEDs) are self-renewing MSCs that reside in the perivascular niche of the dental pulp^7^. These cells are thought to originate from the cranial neural crest, and express early markers of both MSCs and neuroectodermal stem cells. We have previously reported that serum-free conditioned medium from stem cells derived from human exfoliated deciduous teeth (SHED-CM) exerts a protective effect on hepatocytes, and is effective in a rat model of D-galactosamine-induced acute liver failure^8^ and in a murine model of carbon tetrachloride (CCl_4_)-induced liver fibrosis^9^. There have been no reports regarding the effects of SHED-CM in fatty liver models.

In addition, it has recently been reported that abnormalities in intestinal bacteria and increased intestinal permeability promote the progression of NASH^10^. The gut-liver axis refers to the bi-directional relationship between the gut, its microbiota, and the liver^11^. Disturbance of the gut barrier leads to increased portal influx of bacteria or their products into the liver, where they can cause or exacerbate several liver diseases. A high-fat diet alters the microbiome, which impairs the gut barrier and promotes the influx of bacterial products into the portal vein^12^. Since the liver is exposed to high concentrations of lipopolysaccharides (LPS) and other substances, it is most vulnerable to their effects, especially when preconditioned by subclinical pathologies, such as lipid accumulation in hepatocytes^11^.

There are no reports focusing on intestinal permeability in NASH for MSC-based therapies. Appropriate murine models reflecting the complex pathology of NASH are needed to evaluate drugs used to treat lipid accumulation and fibrosis. Mice fed a Western diet and high sugar solution combined with low-dose repeated intraperitoneal CCl_4_ administration for 12 weeks have been reported as a model of NASH with a phenotype similar to that of human NASH^13^. Here, we examined the effects of SHED-CM on the murine NASH model induced by a combination of Western diet (WD) and low doses of intraperitoneal CCl_4_, focusing on the gut-liver axis.

## Methods

### Preparation of SHED-CM

SHEDs were isolated as previously described^14^ at Nagoya University School of Medicine using approved guidelines set by Nagoya University (H-73, 2003). SHEDs were cultured in Dulbecco's modified Eagle's medium (DMEM) with 10% fetal bovine serum. At passages 8–12, SHEDs at 70–80% confluence (2.0 × 10^6^ cells, 10-cm dish) were washed with phosphate-buffered saline (PBS), and the culture medium was replaced with serum-free DMEM. Cells were incubated for 48 h, thereafter, the medium was collected and centrifuged for 5 min at 1,500 rpm. The supernatant was then collected and centrifuged for 3 min at 3000 rpm. The second supernatant was collected and used as the SHED-CM in subsequent experiments. Ethical approval for the use of SHEDs was obtained from the ethics committee of Nagoya University according to the principles of Helsinki Declaration (2015–0278).

### Murine NASH model and Treatment with SHED-CM

Nine-week-old male C57BL/6 J mice from Japan SLC (Shizuoka, Japan) were used for these experiments. Mice were fed a WD containing 21.1% fat, 41% sucrose, and 1.25% cholesterol by weight (Teklad Diets; Envigo, Madison, WI, USA) and a high sugar solution (23.1 g/L d-fructose (Sigma-Aldrich﻿, St. Louis, MO, USA) and 18.9 g/L d-glucose (Sigma-Aldrich))^13^. CCl_4_ (Wako, Osaka, Japan) at a dose of 0.2 μL (0.32 μg)/g of body weight was injected intra-peritoneally once per week during the course of 12 weeks^13^. From week 10 to 12, SHED-CM (0.5 mL) or serum-free DMEM (0.5 mL) was injected once a week through the tail vein. Thirty-six hours after the last treatment, at least six animals per treatment group were sacrificed under deep anesthesia. Liver, ileum, cecal contents, and serum samples were collected and processed for histological, serological, and gene expression analyses. The experimental protocol involving animals was approved by the Institutional Animal Care and Use Committee of Nagoya University (30,311). ﻿Furthermore, all experiments performed in this study were in accordance with the principles of care and use of experimental animals as prescribed by the National Institute of Health and conforms to the ARRIVE guidelines.

### Intestinal permeability

The intestinal permeability was evaluated using the methods previously described^15^. The mice were fasted for 4 h and 4-kDa fluorescein isothiocyanate (FITC)-dextran (Sigma-Aldrich, 20 mg/mL, PBS) was administered orally at a single dose (200 μL). After 4 h, blood from the retrobulbar capillary plexus was sampled into heparinized tubes for 4-kDa FITC-dextran analyses. Plasma was obtained after centrifugation at 2000 × *g* for 5 min. Plasma was diluted 1:5 (v/v) in PBS. Fluorescence was measured spectrophotometrically (SpectraMax iD5; Molecular Devices, Tokyo, Japan) in 96-well plates (excitation: 485 nm, emission: 528 nm). FITC-dextran concentrations were calculated using standard concentrations prepared in PBS ranging from 0 to 12.5 µg/mL 4-kDa FITC-dextran. Emission signals in plasma were calculated by subtraction of those of mice treated with the 4-kDa FITC-dextran from those received PBS.

### ELISA for lipopolysaccharide-binding protein

The lipopolysaccharide-binding protein (LBP) levels in mouse serum were measured using a commercially available enzyme-linked immunosorbent assay (ELISA) kit (Abcam, Cambridge, United Kingdom).

### Real-time quantitative polymerase chain reaction

Total RNA was quantified using a spectrophotometer, and RNA integrity was determined on 1% agarose gels. Reverse transcription reactions were performed with M-MLV Reverse Transcriptase (Invitrogen, Carlsbad, CA, USA) using 0.5 μg total RNA in a 25-μL total reaction volume. Real-time q-PCR was performed using the THUNDERBIRD SYBR qPCR Mix (Toyobo, Osaka, Japan) and the Mx3005P QPCR System Service Program (Agilent, Tokyo, Japan). For each RNA sample, reverse transcription reactions were performed without M-MLV to ensure that the PCR products were not amplified in the downstream reactions. Primers are shown in Supplementary Table. The obtained results for each animal were normalized to hypoxanthine phosphporibosyltransferase or glyceraldehyde-3-phosphate dehydrogenase. Results are expressed relative to the levels in a sham mouse fed a normal diet for 12 weeks.

### Histological and immunohistochemical analyses

The animals were anesthetized and sacrificed 36 h after SHED-CM or DMEM infusion. Formalin-fixed, paraffin-embedded liver samples were cut into 4-μm-thick sections and stained with hematoxylin–eosin (H&E) and Sirius red. NAFLD activity score (NAS) was calculated as the sum of individual scores for steatosis (0–3), ballooning (0–2), and lobular inflammation (0–3)^16^. Sirius red positive area and lipid droplet area were calculated at 20 × magnification using a BZ-9000 microscope (Keyence, Osaka, Japan).

For immunohistochemical analysis, the livers were embedded in optimal cutting temperature compound (Sakura Finetek, Torrance, CA, USA) and cut into 4-μm-thick sections on a cryostat (Leica Biosystems, Wetzlar, Germany). Paraffin-embedded ileal tissue Sects. (4 μm) were deparaffinized and rehydrated in xylene. Antigen retrieval was performed by submerging the sample in 10 mM sodium citrate (pH 6.0) preheated to 98 °C and then heating the sections for another 20 min. The sections were then permeabilized with 0.1% (vol/vol) Triton X‐100 in PBS for 20 min, blocked with 5% (vol/vol) bovine serum albumin for 30 min, and incubated overnight with the following primary antibodies: anti-α-smooth muscle actin (α-SMA) (mouse IgG, 1:500; Abcam), anti-F4/80 (rat IgG, 1:100; Abcam), anti-inducible nitric oxide synthase (iNOS) (rabbit IgG, 1:50; Abcam) for liver or anti-zonula occludens-1 (ZO-1) (rabbit IgG, 1:500; Abcam) for ileum. The following secondary antibodies were used: anti-mouse IgG-Alexa Fluor 488, anti-rabbit IgG-Alexa Fluor 647 or anti-rat IgG-Alexa Fluor 488 and anti-rabbit IgG-Alexa Fluor 488. After counterstaining with 4,6-diamidino-2-phenylindole (DAPI; Sigma-Aldrich), the tissue images were captured using a BZ-9000 microscope. Apoptotic cell death was analyzed using the terminal deoxynucleotidyl transferase 2-deoxyuridine 5-triphosphate nick-end labeling (TUNEL) assay (In Situ Cell Death Detection Kit, Roche Life Science, Geneva, Switzerland). The average number of TUNEL/DAPI—or iNOS/F4/80-positive cells per section was determined by counting 10 random fields in non-overlapping sections at × 20 magnification using the BZ-9000 microscope. The ZO1-stained areas were quantified using a BZ-II analyzer (Keyence, Osaka, Japan). At least three animals per group were examined.

### Triglyceride analyses in liver

Liver samples for lipid extraction were stored at -80 °C until use. Liver lipids were extracted using chloroform/methanol (2:1, v/v) solution according to the Bligh and Dyer method^17^. An aliquot of liver lipid extract was solubilized in 1% Triton X-100 solution and hepatic triglyceride (TG) concentrations were determined by using a Determiner L TG II kit (Hitachi Chemical Diagnostics Systems, Tokyo, Japan).

### Bone Marrow-derived macrophage assay

Analysis of bone marrow-derived macrophages was performed as previously described^18^. Bone marrow cells were isolated from 8-week-old female C57BL/ 6 J mice. They were plated at 2 × 10^6^ cells per 6 cm dish and differentiated into macrophages in DMEM supplemented with 20 ng/mL macrophage colony-stimulating factor (M-CSF) (R&D Systems, Minneapolis, MN, USA) at 37 °C in 5% CO_2_ for seven days. The macrophages were incubated with serum-free DMEM (M0), serum-free DMEM with 20 ng/mL IL-4 (R&D Systems, M(IL-4)), or SHED-CM(M(SHED-CM)) for 24 h, followed by mRNA analysis.

### Caco-2 monolayer assay

Human Caco-2 cells were obtained from the American Type Culture Collection (Manassas, VA, USA). Caco-2 cells were cultured in DMEM supplemented with 10% fetal bovine serum. The cells were incubated in a humidified incubator with 5% CO_2_ at 37 °C and passaged by routine trypsinization. The cells were plated at a density of 5 × 10^4^/cm^2^ on collagen-coated permeable polycarbonate membrane Transwell supports with 0.4 μm pores (Corning, New York, NY, USA) and were grown as monolayers prior to the experiments. ﻿Experiments were performed 14 days after seeding when the cells reached confluence and differentiation. Fresh media was changed every other day in the apical and basolateral compartments of the well until the day of experimentation.

In the experiments, intestinal dysfunction in differentiated Caco-2 monolayers was induced by applying recombinant human TNF-α (10 ng/mL, ﻿R&D Systems) to the basolateral compartment for 6 h after 24 h treatment with human recombinant IFN-γ (20 ng/mL, R&D Systems)^19,20^. After inducing dysfunction in the cell monolayers, the medium in the basolateral chamber was changed to SHED-CM or serum-free DMEM and incubated for 24 h. Internal controls were represented by cells cultured in DMEM without TNF-α or IFN-γ. The barrier function of the cell monolayers was determined by the apical to basolateral flux of 4 kDa FITC-dextran^21^. The apical chamber was filled with Hank's balanced salt solution containing 1 mg/mL FITC-dextran, and concentrations of FITC-dextran in the basolateral chamber were determined spectrophotometrically 2 h later.

### Analysis of gut microbiota

Fecal samples were obtained from the cecum on the day of sacrifice, frozen, and stored at -80 °C. DNA was isolated using the DNeasy PowerSoil Kit (Qiagen, Hilden, Germany). Isolated DNA was amplified to target the V3–4 regions of bacterial 16S rRNA as previously described^22^. PCR products were pooled to construct the sequencing library, which was then sequenced using MiSeq (Illumina, San Diego, CA, USA). We analyzed 16S rRNA gene sequencing data using Greengenes database version 13_8^23^ as a reference.

### Cytokine antibody array

The cytokine antibody array experiments were performed using the RayBio Human Cytokine Antibody Array G Series 4000 (applied arrays; Raybiotech) at Filgen. Using laser scanning, cytokines in SHED-CM and serum-free DMEM were detected by 274-human-cytokine array plates. All scans were performed in duplicate, and data were calculated as the ratio of the level in SHED-CM to that in serum-free DMEM.

### Statistical analysis

The SPSS software package, version 27 (IBM, New York, NY, USA) was used for statistical analyses. Data are presented as bar graphs showing mean ± standard error of the mean. The two groups were compared using the Student’s *t* test or Mann–Whitney test for continuous variables, where appropriate. A *P* value < 0.05 was considered significant.

## Results

### SHED-CM inhibits the development of inflammation and fibrosis in the NASH model

**Figure 1:**
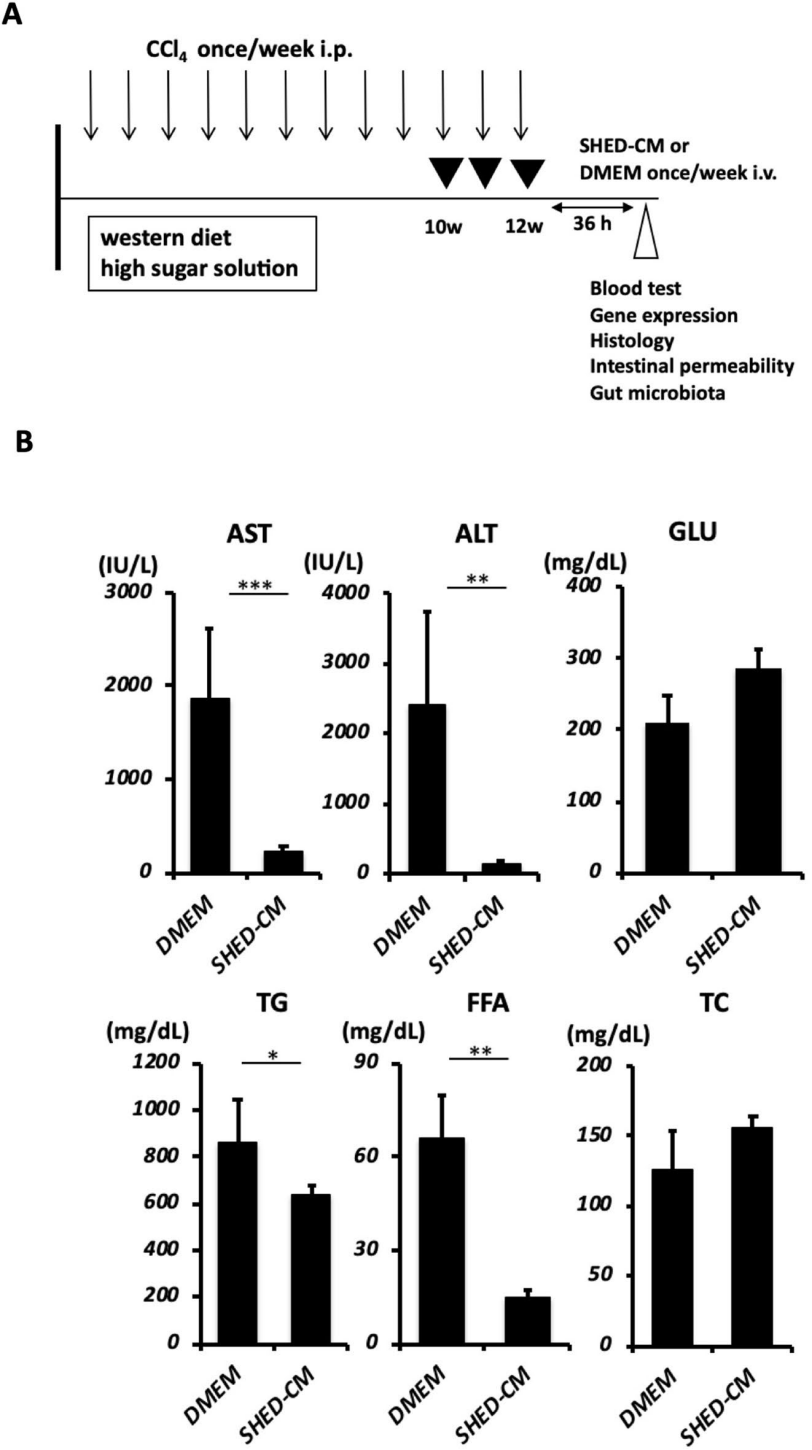
Effects in the peripheral blood of SHED-CM in a murine NASH model. (**A**) C57BL/6 J mice were fed Western diets and a high sugar solution. CCl_4_ was injected intra-peritoneally once per week during the course of 12 weeks. From week 10 to 12, SHED-CM or serum-free DMEM was intravenously administered once a week. Blood, gene expression, histological, intestinal permeability, and gut microbiota analyses were carried out at the indicated time points. (**B**) Quantification of concentrations of AST, ALT, GLU, TG, FFA, and TC in the peripheral blood. Data represent mean ± SEM. *P < .05; **P < .01; ***P < .001. n = 6, DMEM group; n = 8 SHED-CM group. Abbreviations: CCl_4_, carbon tetrachloride; SHED-CM, serum-free conditioned medium from stem cells derived from human exfoliated deciduous teeth; DMEM, Dulbecco's modified Eagle's medium; AST, alanine aminotransferase; ALT, aspartate aminotransferase; GLU, glucose; FFA, free fatty acid; TG, triglyceride; TC, total cholesterol.

**Figure 2:**
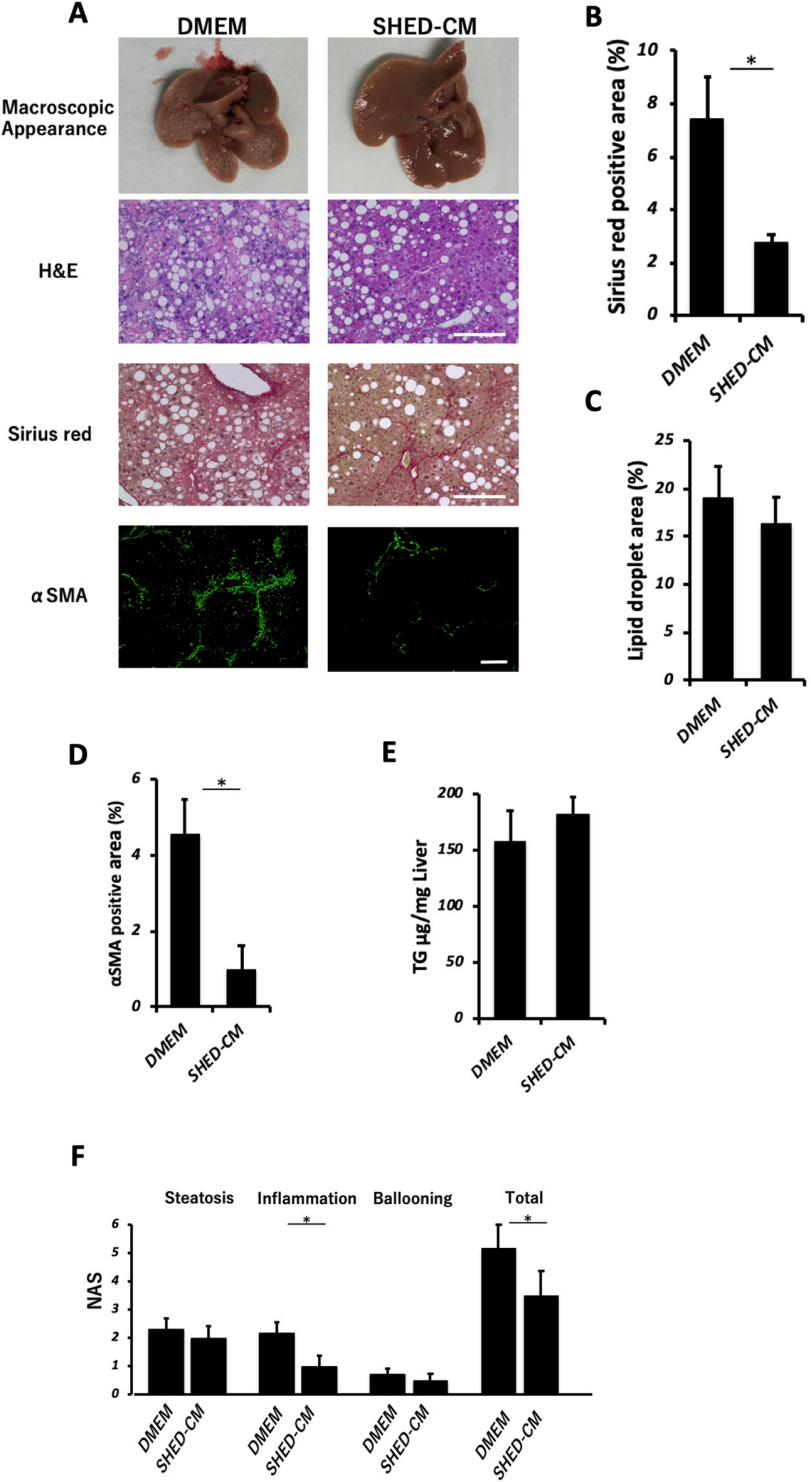
Therapeutic effect of SHED-CM in a murine NASH model. (**A**) Representative images of macroscopic appearance, H&E, Sirius red, and α-SMA immunofluorescent stained livers. Scale bar = 200 μm. (**B**) Quantification of Sirius red-stained areas. (**C**) Quantification of lipid droplet areas according to H&E staining. (**D**) Quantification of α-SMA positive areas according to immunofluorescent staining. (**E**) TG quantification in livers. (**F**) NAS is the sum of the lobular inflammation, ballooning, and steatosis scores according to H&E score. Data represent mean ± SEM. *P < .05. n = 6, DMEM group; n = 8 SHED-CM group. Abbreviations: H&E, hematoxylin and eosin; α-SMA, α-smooth muscle actin; DMEM, Dulbecco's modified Eagle's medium; SHED-CM, serum-free conditioned medium from stem cells derived from human exfoliated deciduous teeth; TG, triglyceride; NAS, non-alcoholic fatty liver disease activity score.

**Figure 3:**
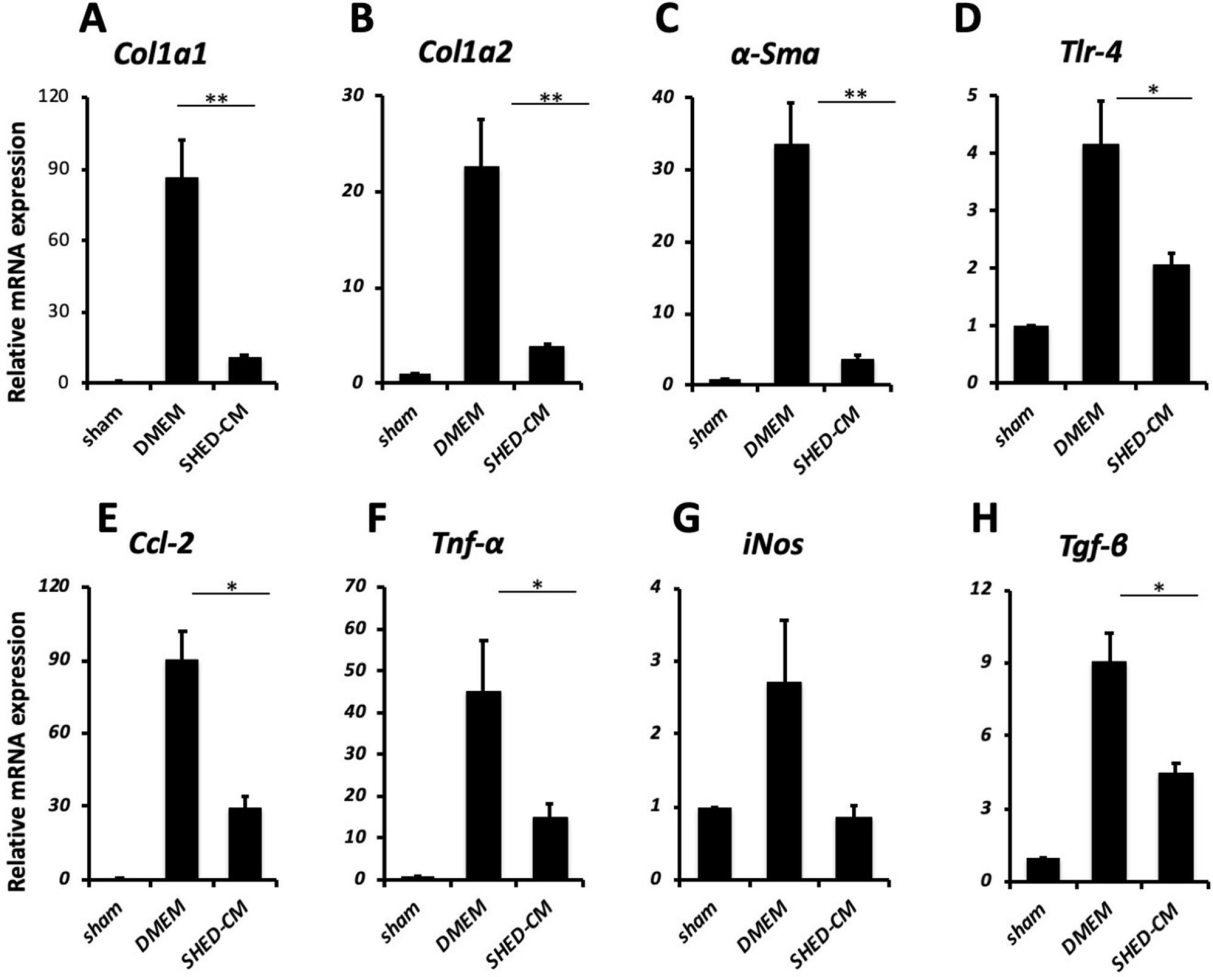
SHED-CM suppresses pro-fibrotic and pro-inflammatory responses. (**A**–**H**) Quantitative polymerase chain reaction analysis of indicated mRNAs. Results are expressed relative to the level in a sham mouse fed a normal diet for 12 weeks. Data represent mean ± SEM. *P < .05; **P < .01; ***P < .001. n = 6, DMEM group; n = 8, SHED-CM group. Abbreviations: DMEM, Dulbecco's modified Eagle's medium; SHED-CM, serum-free conditioned medium from stem cells derived from human exfoliated deciduous teeth; Col1a1, collagen type I alpha 1 chain; Col1a2, collagen type I alpha 2 chain, α-Sma, α-smooth muscle actin; Tlr-4, toll-like receptor 4; Ccl-2, C–C motif chemokine 2; Tnf, tumor necrosis factor; iNos, inducible nitric oxide synthase; Tgf, transforming growth factor.

The SHEDs used in this study exhibited a fibroblastic morphology with a bipolar spindle shape, expressed MSC markers (CD90, CD73, and CD105), but not endothelial/hematopoietic markers (CD34, CD45, CD11b/c, or HLA-DR), and were capable of undergoing adipogenic, chondrogenic, and osteogenic differentiation^14^. There was no significant difference in the cellular survival of the SHEDs after their incubation in serum-free versus serum-containing DMEM (data not shown). Cytokine antibody array analysis revealed 79 proteins in SHED-CM at 1.5-fold or higher levels than those found in the control DMEM background. (Supplementary Fig. 1). Figure 1A shows the animal experiment protocol used in this study. We induced the mouse NASH model as previously reported^13^ and evaluated the therapeutic benefits of weekly administration of SHED-CM or control medium for the last three weeks. Administration of SHED-CM significantly suppressed the elevation of alanine aminotransferase and aspartate aminotransferase levels in peripheral blood. Furthermore, TG and free fatty acid levels were significantly lower in the SHED-CM group than in the DMEM group (Fig. 1B). Morphometric analysis revealed that the positive area with Sirius red staining of the livers was significantly lower in the SHED-CM group (2.75 ± 0.31%) than in the DMEM group (7.43 ± 1.57%, *P* < 0.05) (Fig. 2A,B). In fluorescence immunostaining of liver, the positive area for α-SMA was significantly lower in the SHED-CM group (0.97 ± 0.65%) than in the DMEM group (4.57 ± 0.89%) (Fig. 2A,D). The NAFLD activity score was significantly lower in the inflammation category in the SHED-CM group (1.00 ± 0.19) than in the DMEM group (2.14 ± 0.46, *P* < 0.05), and the total score was also significantly lower in the SHED-CM group (3.50 ± 0.63) than in the DMEM group (5.14 ± 0.99, *P* < 0.05) (Fig. 2F). Conversely, the fat droplet area did not differ significantly between the two groups (Fig. 2C). The accumulation of TG in the liver was not significantly altered by SHED-CM treatment (Fig. 2E). In addition, qPCR analysis showed that SHED-CM treatment significantly suppressed the expression of *Collagen type I (Col1a1 and a2)* and *α-Sma* (Fig. 3A–C). Additionally, we found that the expression of Tlr4, the receptor for LPS, in the liver was significantly suppressed in the SHED-CM group than in the DMEM group (Fig. 3D). *Ccl-2*, *Tnf-α*, and *Tgf-β* mRNAs were also suppressed by SHED-CM treatment (Fig. 3E,F,H). *iNos* mRNA also tended to be suppressed in the SHED-CM group (Fig. 3G).

### SHED-CM inhibits apoptosis of hepatic parenchymal cells and inflammatory macrophage activation in the NASH model

**Figure 4:**
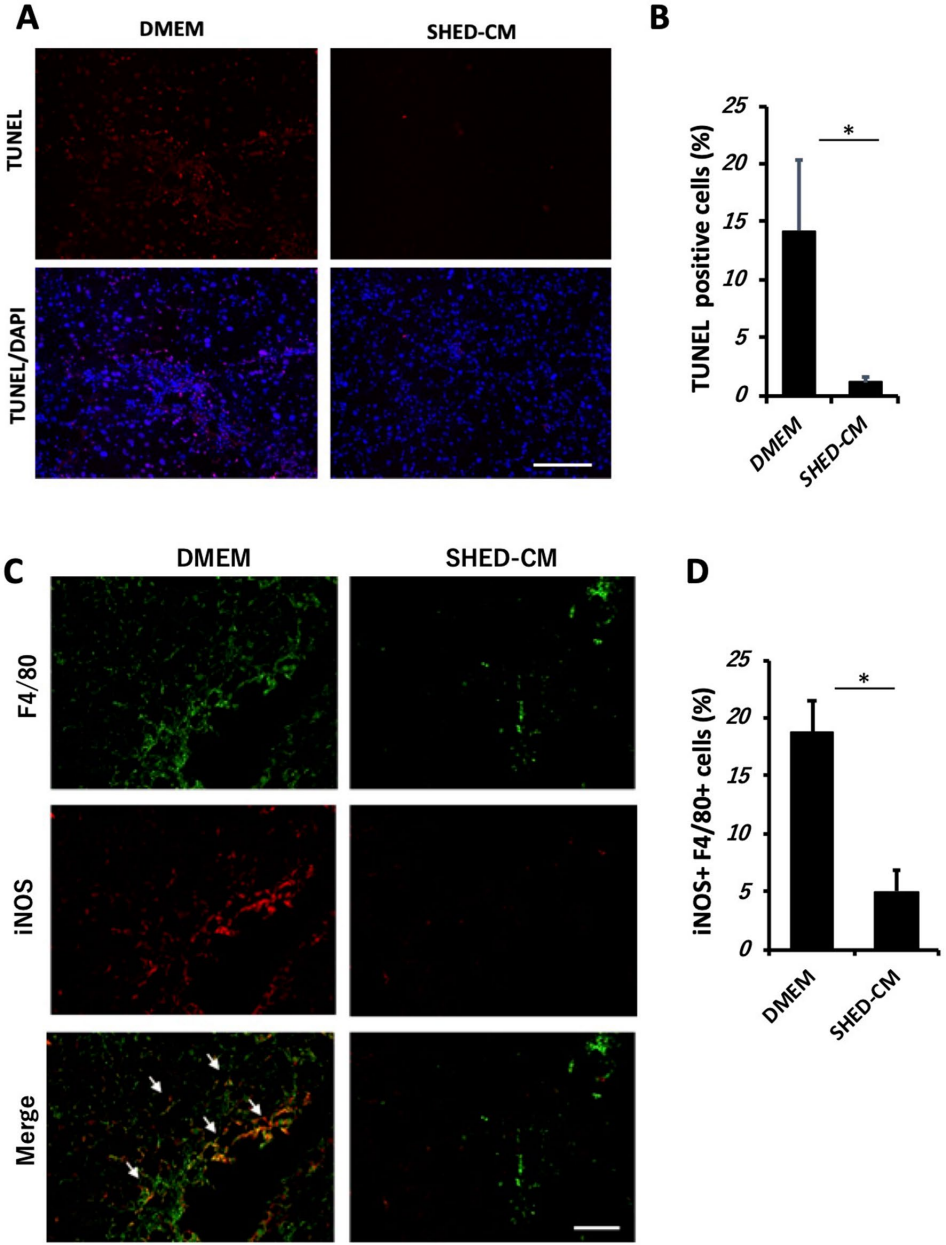
SHED-CM inhibits apoptosis of hepatic parenchymal cells and inflammatory macrophage activation. (**A**) Representative images of TUNEL analysis of liver sections. Scale bar = 200 μm. (**B**) Quantification of TUNEL^+^ / DAPI-stained cells. Data represent mean ± SEM. *P < .05. n = 4, DMEM group; n = 5, SHED-CM group. (**C**) Representative images of immunohistologically stained hepatic macrophages. White arrows indicate that hepatic macrophages express both F4/80 and iNOS. Scale bar = 200 μm. (**D**) Quantification of iNOS^+^ F4/80^+^ cells (%). Data represent mean ± SEM. *P < .05. n = 4 per group. Abbreviations: DMEM, Dulbecco's modified Eagle's medium; SHED-CM, serum-free conditioned medium from stem cells derived from human exfoliated deciduous teeth; TUNEL, terminal deoxynucleotidyl transferase 2’‐deoxyuridine 5’‐triphosphate nick‐end labeling; DAPI, 4’,6‐diamidino‐2‐phenylindole; iNOS, inducible nitric oxide synthase.

Immunofluorescent analysis revealed that the number of TUNEL^+^ cells was markedly reduced in the SHED-CM group compared to the DMEM group. Most TUNEL^+^ cells were located in the parenchymal hepatic lobule (Fig. 4A,B). Histological analysis also revealed that the number of iNOS^+^ F4/80^+^ cells in the SHED-CM-treated mice was significantly lower than in the DMEM-treated mice (Fig. 4C,D).

### SHED-CM suppress the pro-inflammatory and pro-fibrotic reactions of bone marrow-derived macrophages in vitro

**Figure 5:**
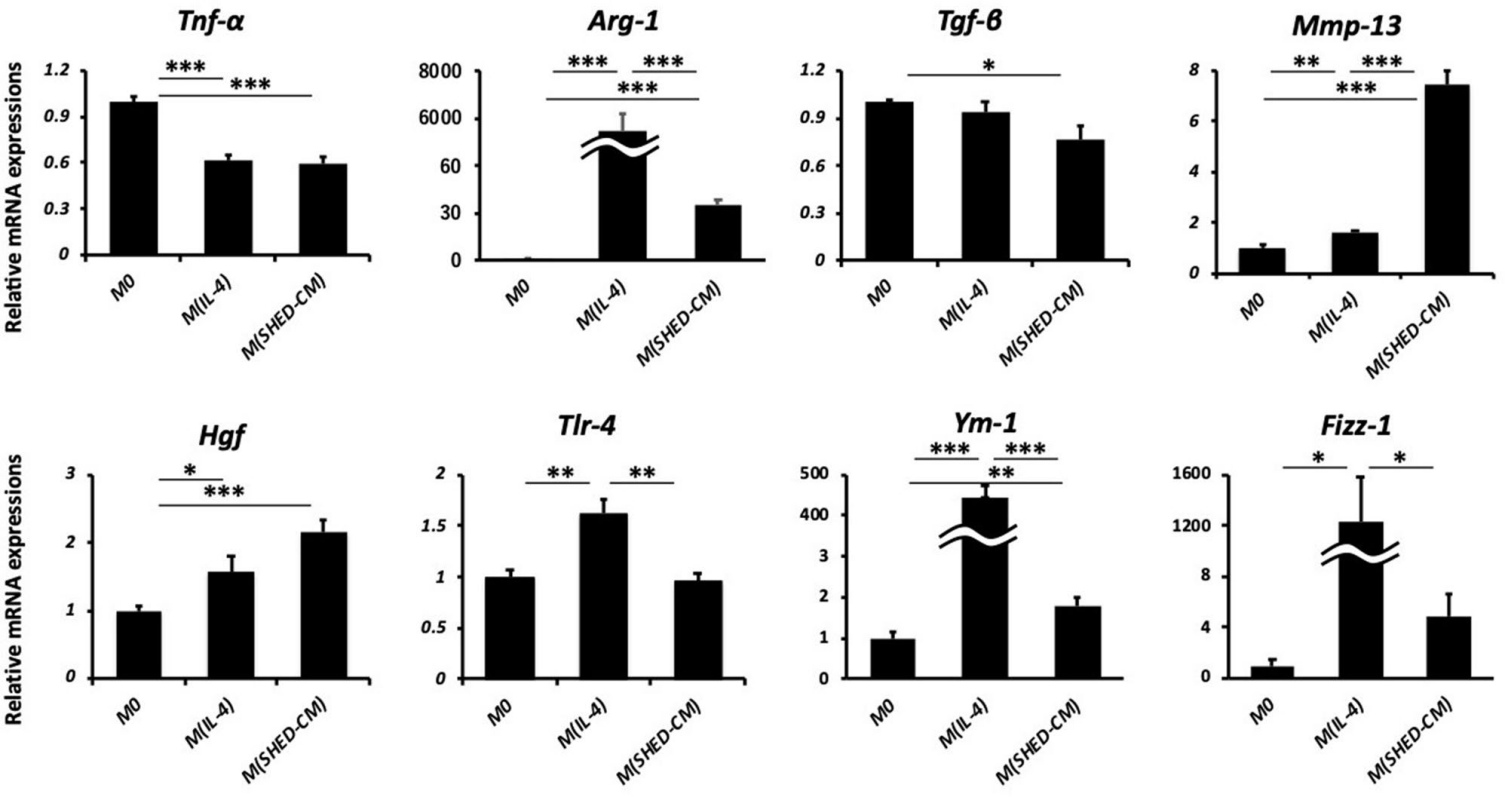
Relative mRNA expression levels of indicated genes in the treated bone marrow-derived macrophages. Data represent mean ± SEM. *P < .05; **P < .01; ***P < .001. Abbreviations: Tnf, tumor necrosis factor; Arg, arginase; Tgf, transforming growth factor; Mmp, matrix metalloproteinase; Hgf, hepatocyte growth factor; Tlr, toll-lile receptor; Fizz-1, found in inflammatory zone 1.

Next, we examined the effects of SHED-CM or DMEM on bone marrow-derived macrophages in vitro. Cells isolated from the bone marrow were subjected to M-CSF for one week to induce macrophages. To examine the characteristics of bone marrow-derived macrophage differentiation stimulated by SHED-CM in vitro, we compared the effect of IL-4, a factor that induces the M2 phenotype, with that of SHED-CM, in addition to DMEM as a control. The expressions of *Tnf*-α and *Tgf*-β were significantly suppressed in SHED-CM-treated macrophages than in the others (Fig. 5). Interestingly, we found that macrophages treated with SHED-CM had some of the characteristics of the M2 phenotype (e.g., *Arg-1* and *Ym-1*) and also showed a high expression of *Mmp-13,* which is a unique phenotype with anti-inflammatory and anti-fibrotic effects.

### SHED-CM protects the intestinal barrier in the NASH model

**Figure 6:**
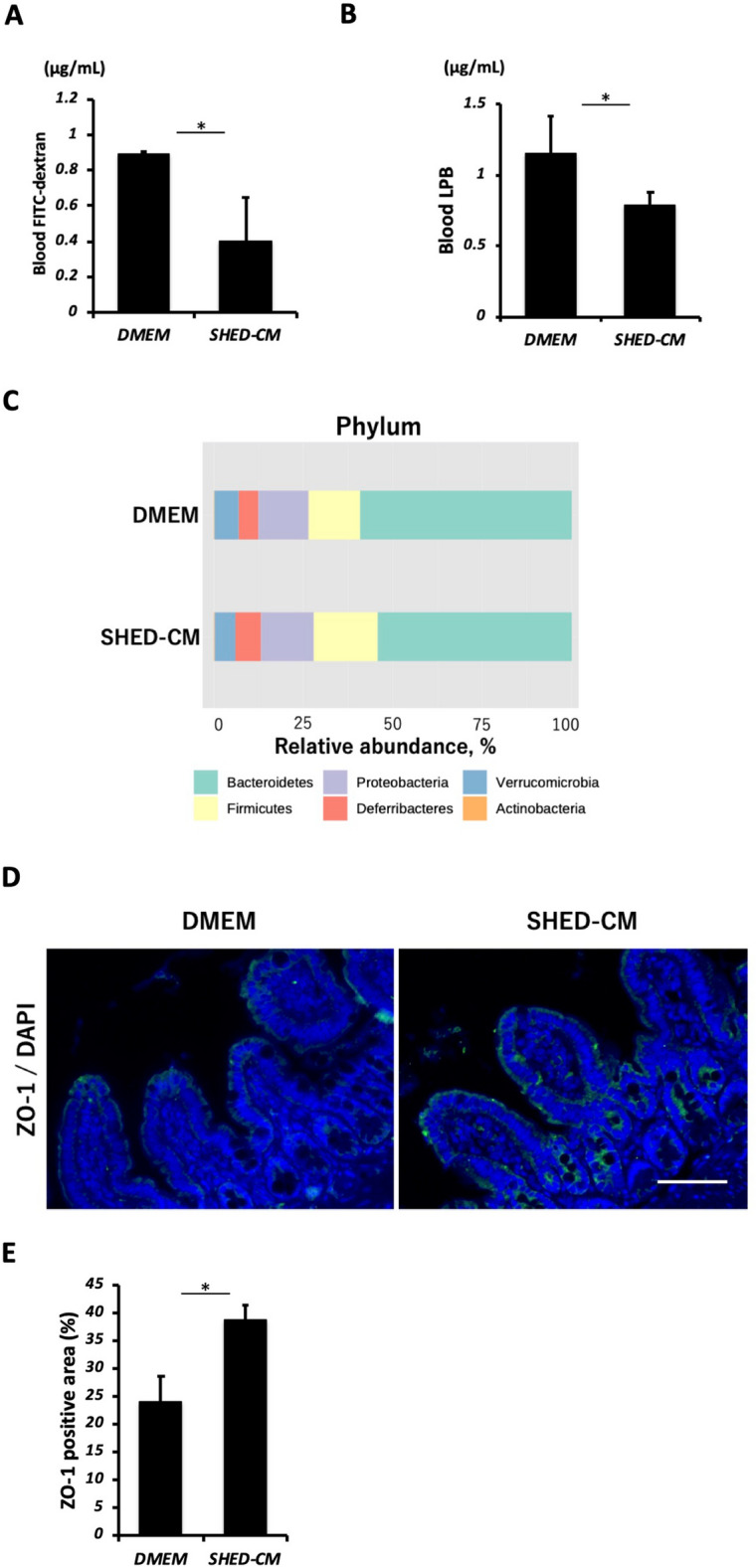
SHED-CM protects the intestinal barrier. (**A**) Concentration of blood FITC-dextran following oral administration to mice. Data represent mean ± SEM. *P < .05. n = 6, DMEM group; n = 8 SHED-CM group. (**B**) Blood LBP concentration. Data represent mean ± SEM. *P < .05. n = 6, DMEM group; n = 8, SHED-CM group. (**C**) Relative abundances of microbiota from cecal contents at the phylum level. n = 6, DMEM group; n = 8 SHED-CM group. (**D**) Representative immunohistological ZO-1 staining images of ileum. Scale bar = 200 μm. (**E**) Quantification of the ZO-1 positive area. Data represent mean ± SEM. *P < .05. n = 3 per group. Abbreviations: FITC, fluorescein isothiocyanate; LBP, lipopolysaccharide-binding protein; DMEM, Dulbecco's modified Eagle's medium; SHED-CM, serum-free conditioned medium from stem cells derived from human exfoliated deciduous teeth; DAPI, 4’,6‐diamidino‐2‐phenylindole; ZO-1, zonula occludens-1.

We next examined intestinal permeability by measuring the fluorescence in peripheral blood following oral FITC-dextran in mice. Increased intestinal permeability was observed in the DMEM group, which was significantly suppressed by SHED-CM treatment (Fig. 6A). The ELISA assay also showed that serum LBP levels were significantly decreased in the SHED-CM group (Fig. 6B). However, there were no differences in gut microbiota either at the genus level (Fig. 6C) or at the genus level between the SHED-CM group and the control group (Supplementary Fig. 2).

To evaluate intestinal epithelial damage, we performed fluorescent immunostaining for the tight junction protein ZO-1 in the ileum and found that the ZO-1 positive area was increased by SHED-CM treatment (Fig. 6D,E).

### SHED-CM restores Caco-2 monolayer dysfunction in vitro

**Figure 7:**
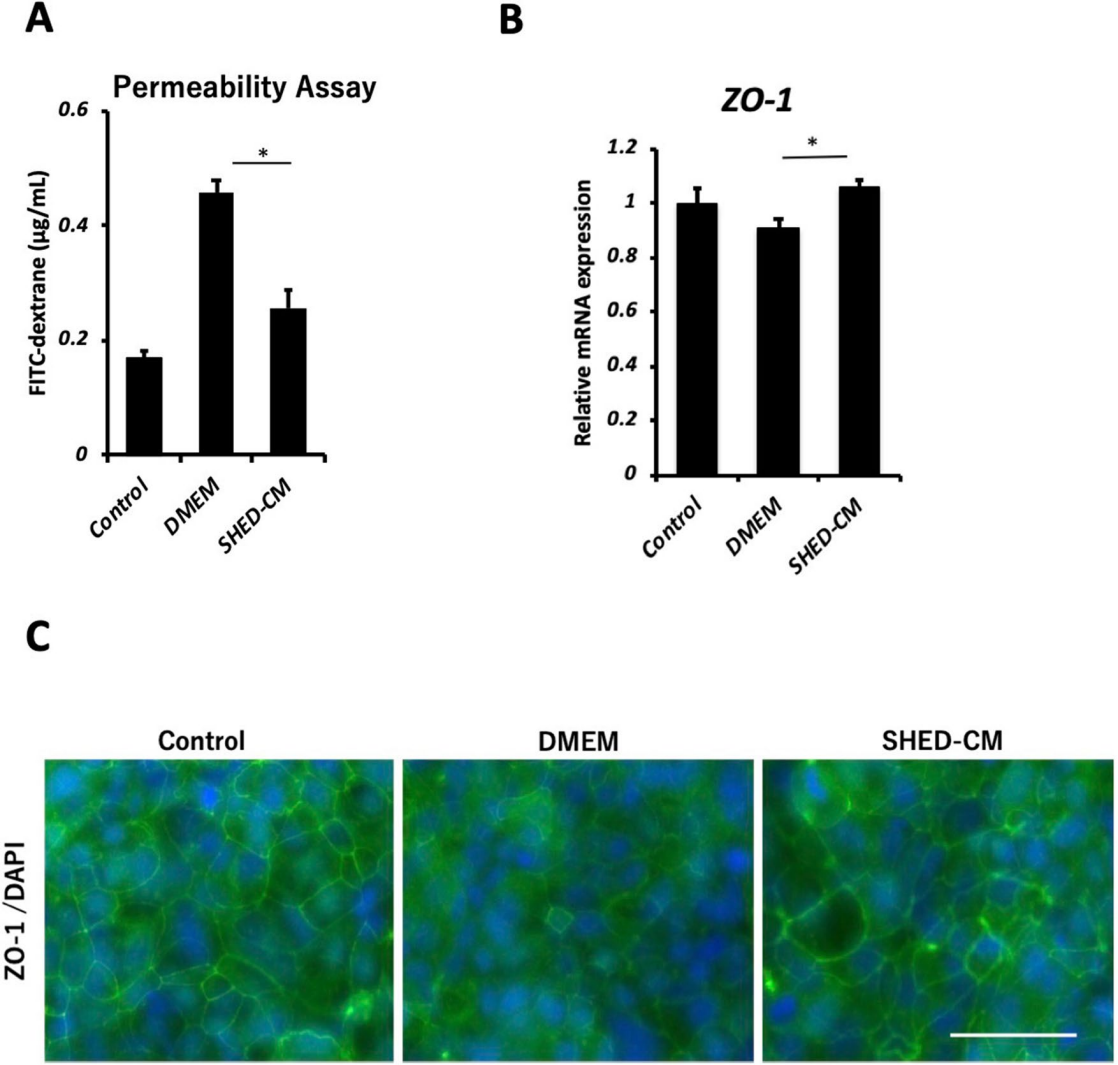
SHED-CM protects functionality of Caco-2 monolayers. (**A**) Quantification of paracellular FITC-dextran flux through Caco-2 monolayers treated with IFN-γ and TNF-α. (**B**) Relative mRNA expression of ZO-1 in Caco-2 cells. Data represent mean ± SEM. *P < .05. (**C**) Representative images of immunofluorescence staining of Caco-2 monolayers. Scale bar = 200 μm. Abbreviations: IFN; interferon; TNF, tumor necrosis factor; FITC, fluorescein isothiocyanate; DMEM, Dulbecco's modified Eagle's medium; SHED-CM, serum-free conditioned medium from stem cells derived from human exfoliated deciduous teeth; ZO-1, zonula occludens-1; DAPI, 4’,6‐diamidino‐2‐phenylindole.

To investigate the effect of SHED-CM on intestinal barrier function, we used Caco-2 monolayers treated with IFN-γ and TNF-α as an in vitro model. The permeability of FITC-dextran, a parameter of epithelial paracellular permeability to uncharged macromolecules, was markedly increased by treatment with IFN-γ and TNF-α. SHED-CM treatment significantly reduced the IFN-γ- and TNF-α-induced increase in paracellular FITC-dextran flux (Fig. 7A). We also found that SHED-CM treatment, after damaging the intestinal barrier, upregulated *ZO-1* expression compared with that in the DMEM group (Fig. 7B). Fluorescence immunostaining of ZO-1 in Caco-2 monolayers is shown in Fig. 7C.

## Discussion

In the present study, we reported on the therapeutic benefits of SHED-CM in a NASH murine model induced by repeated low-dose CCl_4_ administration and WD. This murine model proved to have similar characteristics to human NASH, histologically and transcriptomically^13^. SHED-CM exerted anti-inflammatory and anti-fibrotic effects on the murine NASH model, which could be at least partly due to the protective effect of the intestinal barrier. To the best of our knowledge, this is the first report showing stem cell therapy benefits to the gut-liver axis in a NASH model.

In recent years, stem cell therapies have attracted much attention as a treatment for various liver diseases^24^. We have previously reported that SHED-CM contains several factors involved in hepatocyte protection, anti-apoptosis, angiogenesis, and macrophage differentiation, and has shown efficacy against acute liver failure^8^ and liver fibrosis^9^. Recently, exposure of LPS produced by the gut microbiota to the liver through the portal vein due to increased intestinal permeability (leaky gut) is one of the factors in the progression of NASH^11,25,26^. Bacterial products in the intestine have been shown to be involved in pro-inflammatory and fiber-forming signals via TLRs in the liver^27^. In this study, we showed that SHED-CM protect tight junctions in the intestinal epithelium^28^, maintaining intestinal barrier function. Furthermore, this resulted in a decrease in serum LPΒ concentration, which reflects portal LPS levels, and might have suppressed *Tlr-4* expression in the liver. Although the suppression of the LPS/TLR-4 signaling pathway may be related to the anti-inflammatory effects of SHED-CM on macrophages, at least, part of the therapeutic benefits of SHED-CM on NASH mice may be mediated by protection of the intestinal barrier. Previous studies have suggested that dysbiosis is involved in the development and progression of chronic liver disease^12,29^. We observed that gut microbiota profiles were not altered by SHED-CM treatment. This result suggests that SHED-CM affects the intestinal epithelium directly, not via the change in the gut microbiota profile. Indeed, we have shown that SHED-CM treatment in vitro preserves the function of intestinal epithelial permeability in a Caco-2 monolayer model.

Macrophages are the major phagocytes of the liver and mediate pro-inflammatory and fibrotic effects via TLR-4 in experimental NASH models^30^. In addition to resident macrophages, bone marrow-derived macrophages are recruited in the liver, which is suggested to be involved in inflammation in a murine fatty liver model^31^. It has been previously reported that proteins secreted from SHEDs inhibit the activation of bone marrow-derived macrophages and contribute to the pathogenesis of D-galactosamine-induced acute liver failure in rats^8^. In the present study, we demonstrated that bone marrow-derived macrophages stimulated by SHED-CM have a unique phenotype with a strong *Mmp-13* expression while expressing M2 (anti-inflammatory) markers such as *Arg-1* and *Ym-1 *in vitro. The important role of bone marrow-derived macrophages in SHED-CM-based therapy has been reported previously^32^, and in the NASH model, induction of these unique subsets of macrophages may be involved in the suppression of liver inflammation and fibrosis.

SHED-CM contained several proteins that have been reported to be associated with hepatocyte protection, anti-inflammation, and intestinal epithelial protection^9,33–39^. Specifically, sialic acid-binding Ig-like lectin-9 (Siglec-9) and hepatocyte growth factor (HGF) are uniquely abundant in SHED-CM^8,34^. Hirata et al. demonstrated that HGF plays an important role in the ameliorating effect of SHED-CM on CCl_4_-induced liver fibrosis in a mouse model^9^, suggesting that it could also function as an important ameliorating factor in the CCl_4_-based NASH model employed in this study. Furthermore, Matos et al. showed that it exerts protective effects on the Caco-2 monolayer^40^. Therefore, these previous studies possibly support our hypothesis that HGF also plays an important role in the amelioration of liver fibrosis, exerting a protective effect on the intestinal barrier in our NASH model.

In this study, our NASH murine model showed marked inflammatory cell infiltration with severe steatosis compared with the murine liver fibrosis model established using CCl_4_ only, and it was also observed that SHED-CM ameliorated NASH inflammation by regulating macrophage differentiation. Reportedly, the secreted ectodomain of Siglec-9 (sSiglec-9) induces the M2-like anti-inflammatory phenotype in synergy with monocyte chemoattractant protein-1 (MCP-1)^34,41,42^. In particular, MCP-1 and sSiglec-9 ameliorate rat acute liver failure by inhibiting hepatocellular apoptosis and promoting liver regeneration via the induction of anti-inflammatory/tissue-repairing M2 macrophages^43^. However, the further administration of MCP-1 may not be necessary in the treatment of NASH, given that endogenous MCP-1 levels are elevated in NASH^44^. Therefore, our data suggested that possibly, sSiglec-9 was primarily responsible for the improvement of the disease status in our NASH model. Additionally, a recent study showed that Siglec-9 does not only effect macrophage differentiation, but also exerts a protective effect on intestinal epithelium^39^. However, it is still unclear whether it shows a similar benefitial effect on leaky gut in NASH. Overall, the combined effect of sSiglec-9 and HGF may be involved in the therapeutic benefits associated with SHED-CM in our NASH murine model.

The present study has several limitations that should be addressed. First, the use of human-derived stem cells for murine disease models is partially unclear. We used the murine model that had been proven to have similar characteristics to human NASH, histologically and transcriptomically. We also confirmed the effects of SHED-CM using the human cell line Caco-2. Second, although we showed that SHED-CM treatment reduced TG and free fatty acid concentrations in the peripheral blood, SHED-CM treatment did not significantly change liver fat accumulation. Additional approaches may be necessary to improve fat accumulation in the liver. However, our findings might be clinically meaningful because recent studies have reported that only liver fibrosis, not steatosis, is associated with long-term prognosis in patients with NAFLD in clinical settings^45,46^. Finally, we demonstrated that HGF and sSiglec-9 possibly contribute to the multifaceted therapeutic effects of SHED-CM on NASH models. However, direct evidence in this regard is lacking, hence further investigation is needed.

## Conclusion

We demonstrated that SHED-CM inhibits fibrosis in a murine NASH model via the gut-liver axis, in addition to its protective effect on hepatocytes and inhibition of pro-inflammatory macrophage activation. Our data show that SHED-CM might exert multifaceted therapeutic effects on NASH.

## Supplementary Information

Supplementary Information 1.

Supplementary Information 2.

Supplementary Information 3.

Supplementary Information 4.

## Data Availability

The datasets used and/or analysed during the current study are available from the corresponding author on reasonable request.

## Abbreviations

NAFLD: Non-alcoholic fatty liver disease
NASH: Non-alcoholic steatohepatitis
MSCs: Mesenchymal stem cells
SHEDs: Stem cells from human exfoliated deciduous teeth
SHED-CM: Serum-free conditioned medium from stem cells derived from human exfoliated deciduous teeth
CCl_4_: Carbon tetrachloride
PBS: Phosphate-buffered saline
DMEM: Dulbecco's modified Eagle's medium
FITC: Fluorescein isothiocyanate
LBP: Lipopolysaccharide-binding protein
ELISA: Enzyme-linked immunosorbent assay
H&E: Hematoxylin–eosin
NAS, NAFLD: Activity score
iNOS: Anti-inducible nitric oxide synthase
ZO-1: Zonula occludens-1
DAPI: 4,6-Diamidino-2-phenylindole
TUNEL: Terminal deoxynucleotidyl transferase 2-deoxyuridine 5-triphosphate nick-end labeling
TG: Triglyceride
LPS: Lipopolysaccharide
Siglec-9: Sialic acid- binding Ig-like lectin-9
MCP-1: Monocyte chemoattractant protein-1
