# Supplementary figures and images for: Conditioned medium from stem cells derived from human exfoliated deciduous teeth ameliorates NASH via the Gut-Liver axis
Source: Sci Rep. 2021 Sep 21;11:18778. doi: 10.1038/s41598-021-98254-8 (PMC8455642; doi:10.1038/s41598-021-98254-8)

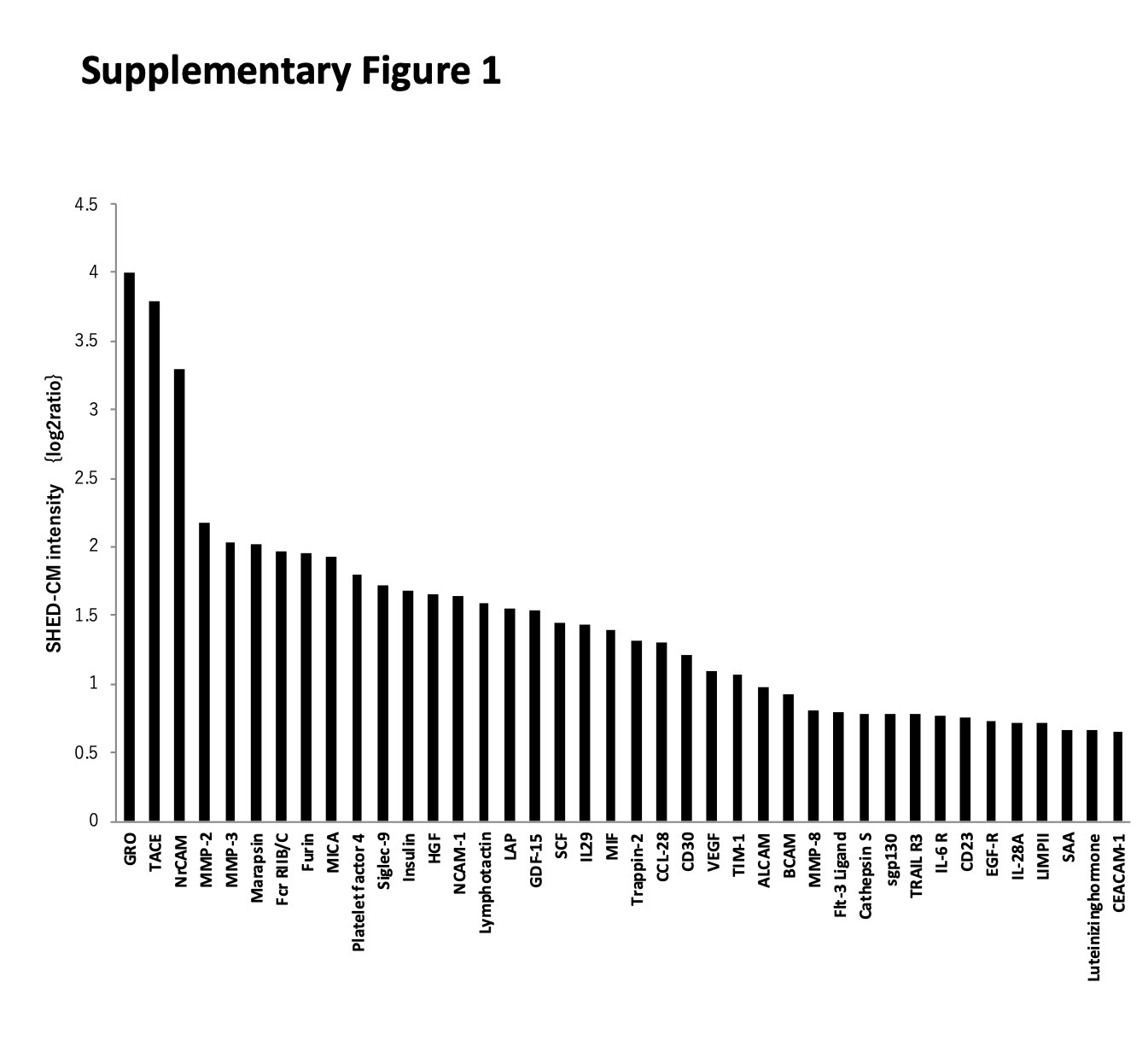

Supplement: Supplementary file 1 — Supplementary Information 1. [file 41598_2021_98254_MOESM1_ESM.jpg]

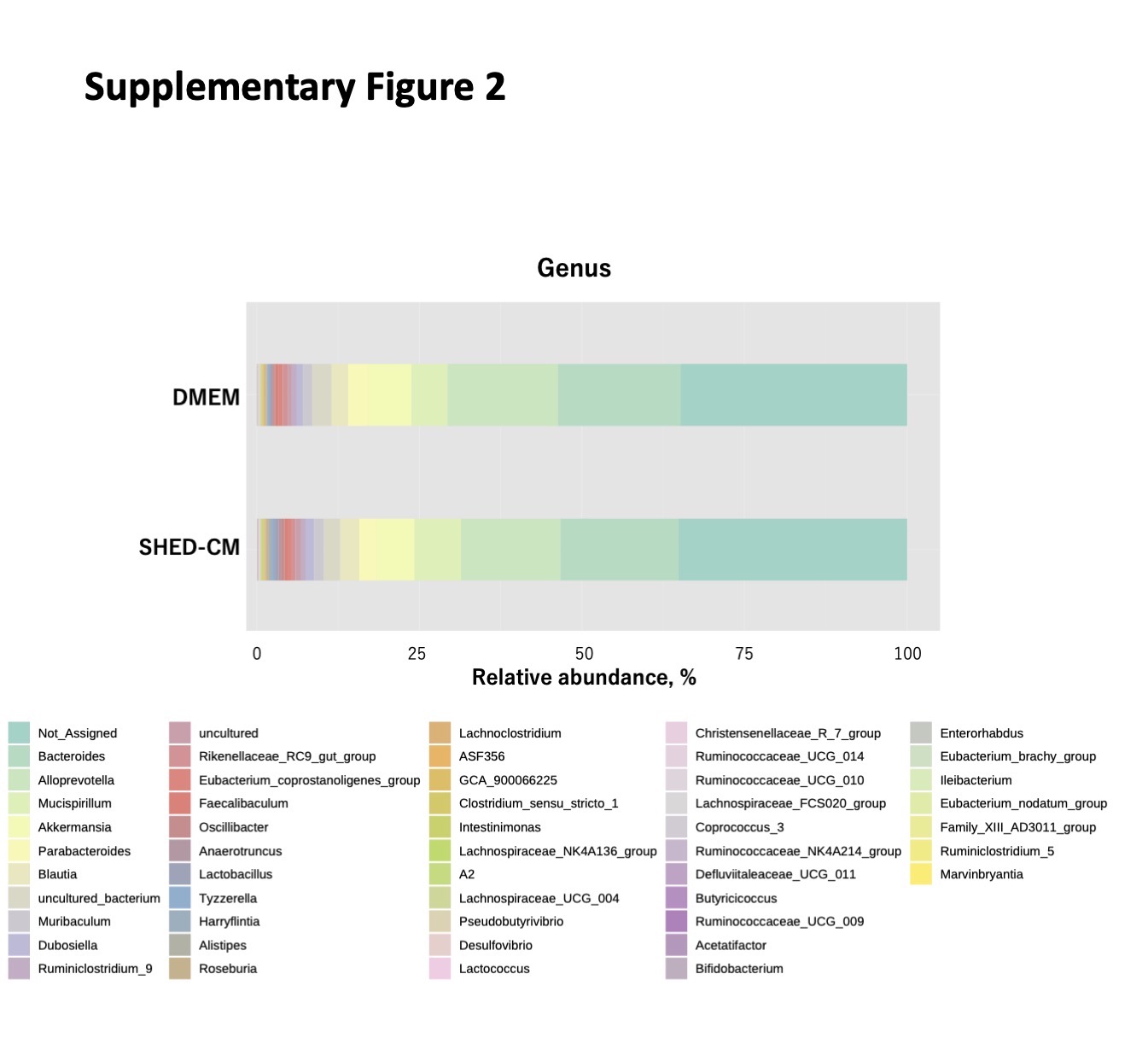

Supplement: Supplementary file 2 — Supplementary Information 2. [file 41598_2021_98254_MOESM2_ESM.jpg]
