# Supplementary material for: Conditioned medium from stem cells derived from human exfoliated deciduous teeth ameliorates NASH via the Gut-Liver axis
Source: Sci Rep. 2021 Sep 21;11:18778. doi: 10.1038/s41598-021-98254-8 (PMC8455642; doi:10.1038/s41598-021-98254-8)
Supplement: Supplementary file 3 — Supplementary Information 3. [file 41598_2021_98254_MOESM3_ESM.docx]

| Mouse Primers |  |  |
| --- | --- | --- |
| Primer | Sequence (forward 5’-3’) | Sequence (reverse 5’-3’) |
| *Col1a1* | GTCCCTGAAGTCAGCTGCATA | TGGGACAGTCCAGTTCTTCAT |
| *Col1a2* | GTGTTCGTGGTTCTCAGGGT | GTCTGAGTGAAGGCTGGGAG |
| *α-Sma* | GCTGTTTTCCCATCCATCGT | GTTGGTGATGATGCCGTGTT |
| *Tlr-4* | TTTATTCAGAGCCGTTGG | CCCATTCCAGGTAGGTGT |
| *CCl-2* | GCAGCAGGTGTCCCAAAGAA | ATTTACGGGTCAACTTCACATTCAA |
| *Tnf-α* | CCCTTTACTCTGACCCCTTTATTGT | TGTCCCAGCATCTTGTGTTTCT |
| *iNos* | AATCTTGGAGCGAGTTGTGG | CAGGAAGTAGGTGAGGGCTTG |
| *Tgf-β* | TACCATGCCAACTTCTGTCTGGG | TGTGTTGGTTGTAGAGGGCAAGG |
| *Ym-1* | TCACTTACACACATGAGCAAGAC | CGGTTCTGAGGAGTAGAGACCA |
| *Fizz-1* | CCAATCCAGCTAACTATCCCTCC | CCAGTCAACGAGTAAGCACAG |
| *Hprt* | TGACACTGGCAAAACAATGCA | GGTCCTTTTCACCAGCAAGCT |
| Human Primers |  |  |
| Primer | Sequence (forward 5’-3’) | Sequence (reverse 5’-3’) |
| *ZO-1* | CGGGACTGTTGGTATTGGCTAGA | GGCCAGGGCCATAGTAAAGTTTG |
| *GAPDH* | GAAGGTGAAGGTCGGAGTC | GAAGATGGTGATGGGATTTC |

Supplementary Table
