# Supplementary material for: Conditioned medium from stem cells derived from human exfoliated deciduous teeth ameliorates NASH via the Gut-Liver axis
Source: Sci Rep. 2021 Sep 21;11:18778. doi: 10.1038/s41598-021-98254-8 (PMC8455642; doi:10.1038/s41598-021-98254-8)
Supplement: Supplementary file 4 — Supplementary Information 4. [file 41598_2021_98254_MOESM4_ESM.docx]

**Supplemental Figure 1**

Factors expressed in SHED–CM at 1.5 times or greater levels than those in serum-free DMEM without cell culture: y-axis, the relative intensity level of factors in SHED–CM compared with DMEM. GRO, chemokine (C–X–C motif) ligand 1; TACE, ADAM metallopeptidase domain 17; NrCAM, neuronal cell adhesion molecule; MMP, matrix metalloproteinase; MICA, MHC class I polypeptide-related sequence A, HGF; hepatocyte growth factor, NCAM-1; neural cell adhesion molecule 1; LAP, latency-associated peptide; GDF-15, growth differentiation factor 15, SCF, stem cell factor; IL29, interleukin 29; MIF, macrophage migration inhibitory factor; CCL 28, C-C Motif Chemokine Ligand 28; CD, cluster of differentiation, VEGF; vascular endothelial growth factor; TIM-1, T-cell immunoglobulin and mucin domain 1; ALCAM, activated leukocyte cell adhesion molecule; BCAM, basal cell adhesion molecule; Flt-3 Ligand, Fms-related tyrosine kinase 3 ligand; sgp130, soluble glycoprotein 130; TRAIL R3, tumor necrosis factor related apoptosis inducing ligand 3; IL-6 R, interleukin 6 receptor ; EGF-R, epidermal growth factor receptor; IL-28A, interleukin 28A; LIMPII, lysosomal integral membrane protein II; SAA, serum amyloid A; CEACAM-1, carcinoembryonic antigen-related cell adhesion molecule 1.

**Supplemental Figure 2**

Relative abundance of microbiota from cecal contents at the genus level. n = 6, DMEM group; n = 8, SHED-CM group.
